# Mouse Model of Fast-Channel Genetic Myasthenic Syndrome Carrying *Chrne* p.P141L Mutation

**DOI:** 10.3390/biom16070931

**Published:** 2026-06-23

**Authors:** Richard G. Webster, Susan Maxwell, Yin Y. Dong

**Affiliations:** Nuffield Department of Clinical Neurosciences, University of Oxford, Oxford OX3 9DS, UK

**Keywords:** fast channel genetic myasthenic syndrome, acetylcholine receptor, mouse model

## Abstract

Fast-channel genetic myasthenic syndromes (FCGMSs) are caused by genetic variants in muscle nicotinic acetylcholine receptor (AChR) subunits that reduce channel open times and impair neuromuscular transmission. Among these, the *CHRNE* p.P141L variant (εP141L) is associated with particularly severe disease. Here, we characterized a knock-in mouse model harboring the homologous p.P141L variant in *Chrne* (εP141L)—C57BL/6J-Chrne^em1H^/H made by the MRC GEMM program. Homozygous mutant mice fail to thrive, with early lethality (median survival of 16 days), closely recapitulating the severity observed in patients. Despite a preserved neuromuscular junction (NMJ) morphology and robust AChR expression, electrophysiological analyses revealed marked reductions in miniature and evoked endplate potential amplitudes and areas, accompanied by prolonged depolarization kinetics (contrary to expectations for AChR with reduced open times) and increased quantal content, indicative of impaired post-synaptic function with compensatory pre-synaptic adaptation. Notably, disease severity exceeded that of *Chrne* null mice, likely through competition with more functional g-subunit-containing fetal AChRs. Consistent with this, crossing εP141L mice with *CHRNG*-expressing mice provided little survival benefit. These findings demonstrate that dysfunctional AChR incorporation is more deleterious than receptor absence and highlight the critical role of subunit composition in sustaining neuromuscular transmission. Pharmacological enhancement of pre-synaptic release with 3,4-diaminopyridine partially improved synaptic parameters. In addition, the AChR-positive allosteric modulator DC-98 modestly improved neurotransmission. Thus, this mouse model provides a faithful platform for mechanistic studies and therapeutic development in FCGMS.

## 1. Introduction

Neuromuscular transmission is a highly complex process essential for initiation and control of muscular contraction, which is essential for all voluntary movement [1]. This activity relies on the precise location and function of many synaptic proteins at the neuromuscular junction (NMJ). The key signal transduction molecules here are muscle nicotinic acetylcholine receptors (AChRs), which are pentameric ligand-gated ion channels (pLGICs) that receive the chemical acetylcholine (ACh) signal from the motor neuron and translate it into an electrical signal, initiating post-synaptic depolarization. Their precise kinetic functioning is fundamental to effective, robust and reproducible neurotransmission [2].

Genetic myasthenic syndromes (GMSs), also known as congenital myasthenic syndromes (CMSs), are a heterogenous group of syndromes characterized by fatigable muscle weakness, caused by disruption of neurotransmission at the neuromuscular junction (NMJ) [3,4]. GMS has a reported prevalence of between 1.8 and 22.2 per million depending on the population [5,6,7,8,9,10], which is likely an underestimate due to difficulties in diagnosis.

Indeed, 40 genes have been identified that can carry genetic variants that cause disruption of neuromuscular transmission [11]. GMS is most commonly caused by genetic variants in the genes that encode the muscle AChR, which can lead to a reduction in muscle AChR expression at the NMJ, or changes in AChR function. Genetic variants that increase AChR function resulting in elongated channel openings cause slow channel GMS (SCGMS), whereas those that reduce AChR function and ultimately lead to abbreviated AChR opening are called fast-channel GMS (FCGMS).

FCGMS is defined by kinetic alteration in the AChR that results in reduced signaling at the NMJ due to significant shortening of AChR activation when ACh is bound to the receptor [12]. This distinguishes FCGMS from the AChR-deficiency syndromes: whereas AChR-deficiency variants reduce the number of functional receptors expressed at the endplate, fast-channel variants leave receptor numbers largely intact but impair receptor gating, so that each binding event produces a briefer channel opening and a smaller post-synaptic response. The consequent loss of the safety margin for neuromuscular transmission can be profound, and the clinical phenotype depends critically on how severely a given variant disrupts receptor function. The most common and severe form of FCGMS in the UK is caused by the *CHRNE* p.P141L variant (εP141L, previously known as εP121L without the signal peptide sequence, with a minor allele frequency of 0.0000357 (GnomAD_Exomes)), either present as a homozygous variant, or in combination with a *CHRNE* variant that causes AChR deficiency at the NMJ [13]. This FCGMS variant was first described in 1996 and has been extensively studied to describe the kinetic disruption to its functioning [14]. It effectively disrupts one of the ACh-binding sites at the α-ε subunit interface of the AChR pentamer, resulting in a 280-fold reduction in receptor activity. FCGMS patients with the εP141L variant are characterized by severe generalized fatigable muscle weakness, as well as breathing and feeding difficulties. Untreated patients do not survive childhood; current management of FCGMS is entirely symptomatic and is directed at increasing the amount of ACh available in the synaptic cleft, such as cholinesterase inhibitors like pyridostigmine and 3,4-diaminopyridine (3,4-DAP). In addition, β2-adrenergic agonists such as salbutamol are used as adjuncts [15]. However, as the εP141L AChR is so inactive, increasing ACh availability has a very limited effect on the disease phenotype, and patients optimized on the best current treatments remain severely weak (average quantitative myasthenia gravis score, QMG, of 25/39) and can still die in childhood [13]. None of the therapies target the primary kinetic abnormality, and no disease-modifying therapy is currently available, underscoring the need both for new treatment approaches and experimental systems in which to evaluate them.

The severity of AChR-related syndromes, and the extent to which they are reproduced in mice, is further shaped by the developmental switch in receptor subunit composition. During fetal development the muscle AChR incorporates a γ-subunit in place of the adult ε-subunit, generating a fetal receptor with distinct kinetic properties [14]. In humans, persistent low-level expression of *CHRNG* allows continued formation of these γ-subunit-containing fetal receptors, which can sustain residual neuromuscular transmission and survival even in patients who express little or no functional ε-containing adult AChR [13]. In mice, by contrast, *Chrng* expression is switched off a few days after birth [15], so any compensation provided by fetal receptors is progressively lost over the first post-natal weeks. The amount and persistence of γ-containing fetal AChR is therefore an important determinant of disease severity and of survival in mouse models of AChR-related syndromes and is central to interpreting the consequences of a fast-channel variant in vivo. Despite extensive in vitro characterization of the εP141L receptor, no animal model carrying this variant (or any other FCGMS variant) has previously been available. Thus, it has so far been impossible to test the effects of the variant on neuromuscular transmission and survival in an intact model organism, or to assess their response to candidate therapies, which is necessary for preclinical trials.

Here, we describe the characterization of a mouse model of this GMS variant by the insertion of εP141L into mouse *Chrne* (as part of the MRC GEMM program). With a faithful recapitulation of this form of GMS in a mouse model, we hope to create an effective translational platform for developing novel therapies for FCGMS.

## 2. Materials and Methods

### 2.1. Generation of Mutant Mice

The C57BL/6J-Chrne^em1H^/H mice were obtained from the Mary Lyon Centre at MRC Harwell, which is the UK node of the European Mouse Mutant Archive (EMMA) (https://www.infrafrontier.eu/emma/strain-details/?q=16095, accessed on 10 June 2026) [16]. See Supplementary Materials for more details on how the model was generated. The AChR-deficiency model mice used to generate transgenic mice with ε knockout and CHRNG expression were previously described.

Various genotypes of C57BL/6J-Chrne^em1H^/H mice are simplified as follows: homozygous (ε^P141L^/ε^P141L^), also called fast channel model mice; heterozygous (ε^+^/ε^P141L^) and wildtype C57Bl/6J (ε^+^/ε^+^); and *Chrne* knockout mice (ε^−^/ε^−^) and mice expressing *CHRNG* as (+hg). The crossing of AChR-deficiency mice with C57BL/6J-Chrne^em1H^/H mice produced mice with a slight variation in background strain, which are referred to as (ε^P141L^/ε^P141L^ (mixed strain)).

### 2.2. Genotyping

Genomic DNA was extracted from mouse ear clip biopsies by overnight incubation at 55 °C in lysis buffer containing proteinase K, followed by heat inactivation at 95 °C for 45 min. The lysate was used directly as a template for PCR.

For genotyping of C57BL/6J-Chrne^em1H^/H mice, PCR amplification was performed using primers: forward 5′-**GCAGGTGTAGGAATCCTCCG**-3′ and reverse 5′-**GGCAGCAAGAAACCTGTGCC**-3′. Thermal cycling conditions were: 95 °C for 1 min; 35 cycles of 95 °C for 30 s, annealing at 60 °C for 30 s, and extension at 72 °C for 1 min; followed by a final extension at 72 °C for 5 min.

PCR products were subsequently digested with BseYI for 2 h at 37 °C. The restriction site (CCCAGC) is present in the wildtype allele but absent in the mutant allele. Digested products were resolved on a 3% agarose gel.

The mutant allele yielded an undigested single band of 510 bp, whereas the wildtype allele produced two fragments of 245 bp and 265 bp. Heterozygous animals displayed all three bands.

### 2.3. NMJ Imaging

Dissected mouse hemidiaphragms were pinned out onto sylgard and fixed in 3% paraformaldehyde in PBS for 60 min. For neurofilament and synaptophysin staining, the fixed mouse hemidiaphragms were washed in PBS (6 × 10 min), permeabilized for 1 h with 0.3% Triton-X 100 (Merck Life Science, Dorset, UK, Cat. No T8787) in PBS followed by a tissue wash in PBS (3 × 10 min), and then blocked with 4% bovine serum albumin (BSA) in 0.5%Tween/PBS for 1 h at room temperature under gentle agitation. Samples were incubated overnight at 4 °C in PBS containing 4% BSA/PBS-T, a 1:500 dilution of rabbit anti-neurofilament heavy chain (Cell Signalling Technology, Leiden, The Netherlands, Cat. No 2837T), and a 1:500 dilution of rabbit anti-synaptophysin (Abcam Inc., Waltham, MA, USA, Cat. No ab32127). The following day, diaphragms were washed in PBS (3 × 20 min). The neurofilament and synaptophysin staining were visualized by incubating samples for 2 h at room temperature in 4% BSA/PBS containing 488-Alexa Fluor^®^ goat anti-rabbit secondary antibody (1:100) (ThermoFisher Scientific, Loughborough, UK, Cat. No A11008), while 594 nm α-bungarotoxin (1:150) (Invitrogen, Waltham, MA, USA, Cat. No B13423) was added after 1 h for visualization of AChRs. The diaphragms were extensively washed in PBS to reduce background staining prior to being mounted onto microscope slides (Avantor VWR, Lutterworth, UK, Cat. No 631-0108) using hard-set confocal matrix (Micro tech Lab).

Fluorescence was observed and Z-stacks of neuromuscular junctions were captured using a Zeiss LSM 780 upright confocal microscope (25× magnification) with ZEN 2.3 LSM software (Zeiss Microscopy, Edinburgh, UK). For assessment of NMJ size, fragmentation, area, and nerve terminal registration, optimized settings for each image were used. Between 3 and 5 Z-stacks were obtained per diaphragm. At least 10 NMJs were analyzed from each sample using ImageJ software (ver 1.54). Imaging and analyses took place in a blinded fashion.

### 2.4. Phrenic Nerve-Diaphragm Electrophysiology

Following euthanasia of P15 to P17 mice, phrenic nerve/hemi-diaphragm preparations were dissected and bathed in Krebs solution (118 mM NaCl, 4.7 mM KCl, 1.2 mM MgSO_4_·7H_2_O, 1.2 mM KH_2_PO_4_, 24.9 mM NaHCO_3_, 10 mM d-glucose, pH 7.4) containing 2.5 mM CaCl_2_ and bubbled with 95% O_2_/5% CO_2_. μ-Conotoxin GIIIB (2.5 μM, Peptide Institute Inc., Osaka, Japan) was added to the bath for 30 min to block muscle contractions. The excess unbound toxin was washed out before recordings started. Recordings were made at 22–23 °C.

The phrenic nerve was pulled into a suction electrode, which was coupled to a pulse generator, with an associated stimulus isolation unit (GRASS instruments S48 square pulse stimulator, A-M systems, Sequim, WA, USA). Recording electrodes were connected to an Axoclamp 900A amplifier (Molecular Devices, San Jose, CA, USA). Data signals passed through a Humbug 50 Hz noise eliminator (Quest Scientific via Digitimer, Letchworth, UK). Signals were continuously digitized at a 10 kHz sampling rate and filtered at 2 kHz, using the Axon Digidata 1440A interface, controlled by pClamp 10 software (Molecular Devices). Depolarizations at the endplate were recorded intracellularly using a single borosilicate glass micropipette electrode. A two-electrode voltage clamp (TEVC) was used to record membrane current. Electrodes were pulled by a programmable P-97 microelectrode puller (Sutter Instruments, Novato, CA, USA) and filled with 3M KCl (10–30 MΩ). The recording electrode/s was positioned above endplate regions, as visualized by a stereomicroscope (Olympus BX51WI) under micromanipulator control (Scientifica, Uckfield, UK).

Impalement of individual muscle fibers adjacent to the neuromuscular junction (endplate) was indicated by a fast rise time of miniature endplate potentials (mEPPs), defined as less than 2 ms. mEPP parameters were measured on averaged traces obtained from >20 recorded events and EPPs were averaged from 20 events in a 1 Hz train. mEPP and EPP amplitudes were adjusted to a standardized membrane potential of –80 mV, and quantal content was calculated accounting for non-linear summation by a direct method using adjusted EPP amplitude [17,18]. Recordings in the presence of DC-98 and 3,4-DAP were made following at least 15 min of incubation with the compound.

### 2.5. Statistics

For electrophysiological data, non-parametric statistical comparisons were made without assumption of data normalcy due to relatively low numbers of independent preparations used. Multi-group data were compared by Kruskal–Wallis ANOVA followed by Dunn’s multiple comparison test; for comparison of two groups, Mann–Whitney tests were performed (GraphPad Prism, ver 11.0.2). Data are presented as mean ±95% confidence interval. The data points plotted are the mean of multiple replicate measurements from individual preparations. The biological unit (*n*) is the number of mice studied. Kaplan–Meier survival curves were compared by the logrank (Mantel–Cox) test within Graphpad Prism. In the figures, statistical significance is indicated; ns *p* ≥ 0.05; * *p* < 0.05; ** *p* < 0.01; *** *p* < 0.001; **** *p* < 0.0001.

## 3. Results

### 3.1. Growth and Survival

**Figure 1:**
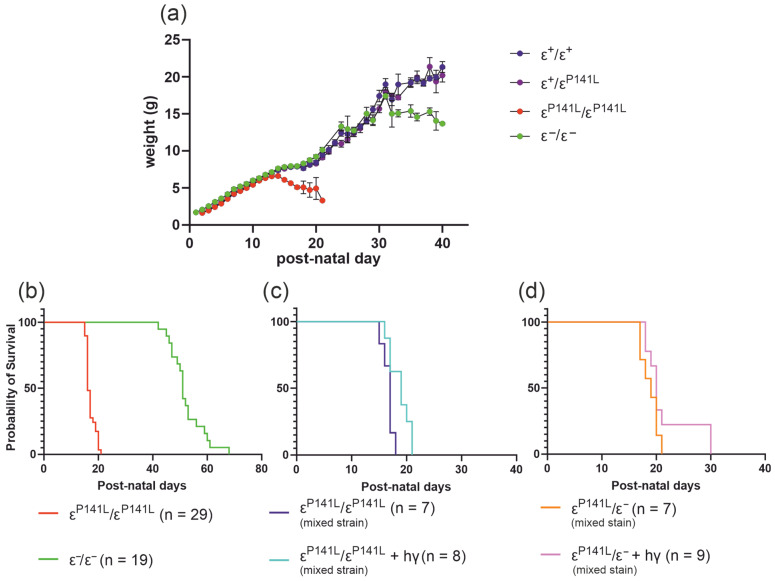
Growth and survival of fast-channel mice. Panel (**a**) shows weights of animals recorded from post-natal day 1. Weights were recorded daily from ε^P141L^/ε^P141L^ mice (*n* = 24), ε^+^/ε^P141^ mice (*n* = 41), ε^+^/ε^+^ mice (*n* = 24), and ε^−^/ε^−^ mice (*n* = 11). Panel (**b**) shows survival curves for ε^P141L^/ε^P141L^ and ε^−^/ε^−^ mice; median survival was significantly different (*p* < 0.0001, Hazard Ratio (HR): 4.1; 95% CI: 2.2–7.8) at 16 and 51 days respectively (*n* = 29 and 19). Panel (**c**) shows survival curves for ε^P141L^/ε^P141L^ (mixed strain) and ε^P141L^/ε^P141L^ + hg (mixed strain); median survival increased but was significantly improved (*p* = 0.02, HR: 0.39; 95% CI: 0.1–1.4) at 17 and 19 days respectively (*n* = 6 and 8). Panel (**d**) shows survival curves for ε^P141L^/ε^−^ and ε^P141L^/ε^−^ + hg mice respectively. Median survival was not significantly increased (*p* = 0.151, HR: 0.56; 95% CI: 0.2–1.6) at 19 and 20 days. Kaplan–Meier curves were tested for significant differences by the logrank (Mantel–Cox) test.

Model mice were generated by breeding C57BL/6J-Chrne^em1H^/H male with female mice, which are heterozygous for the *Chrne* p.P141L variant (ε^+^/ε^P141L^). Homozygous fast-channel model mice (ε^P141L^/ε^P141L^) were born at the expected ratio at 26% of all births. Daily monitoring of weight (Figure 1a) showed normal weight gain until ~PND14, at which time mice began to exhibit reduced voluntary movement, failed to thrive, stopped gaining weight and began to lose weight such that the humane endpoint (loss of >15% max weight) was reached at a maximum of PND21, before weaning age. At PND15 and onwards, model mice weight was significantly reduced compared to wildtype (PND15: 6.1 g vs. 7.8 g, respectively; *p* < 0.001; unpaired *t*-test with Holm–Sidak multiple comparison correction). Median survival was 16 days (Figure 1b).

Mice with *Chrne* expression knocked-out (ε^−^/ε^−^) had a median survival of 51 days but showed no weight loss until ~40 days. In this FCGMS model, weight loss and death began sooner compared to *Chrne* knockout mice. Furthermore, in ε^−^/ε^−^ mice, insertion of *CHRNG* (ε^−^/ε^−^ + hg) allowed normal mouse survival up to 2 yrs, despite still having compromised neuromuscular transmission and significant weakness in comparison to WT ε^+^/ε^+^ mice [19]. However, for fast-channel mice, inclusion of human *CHRNG* (ε^P141L^/ε^P141L^ + hg) only improved median survival from 17 to 19 days (Figure 1c), and even removing one copy of ε^P141L^ did not significantly enhance the survival of ε^−^/ε^P141L^ + hg mice (Figure 1d). However, these changes are small, and interpretation must be tempered by the relatively low numbers of animals studied.

### 3.2. NMJ Imaging

Imaging of the diaphragm muscle stained with fluorescent α-bungarotoxin showed AChR expression at the NMJ of ε^P141L^/ε^P141L^ mice, comparable to an age-matched WT diaphragm. This contrasted with the weak fragmented staining seen in AChR-deficiency ε^−^/ε^−^ mice (see Figure S1 for example images and quantification), indicating that in this model (even though only low numbers were examined), NMJ AChR staining does not predict model severity.

### 3.3. NMJ Neurotransmission

**Figure 2:**
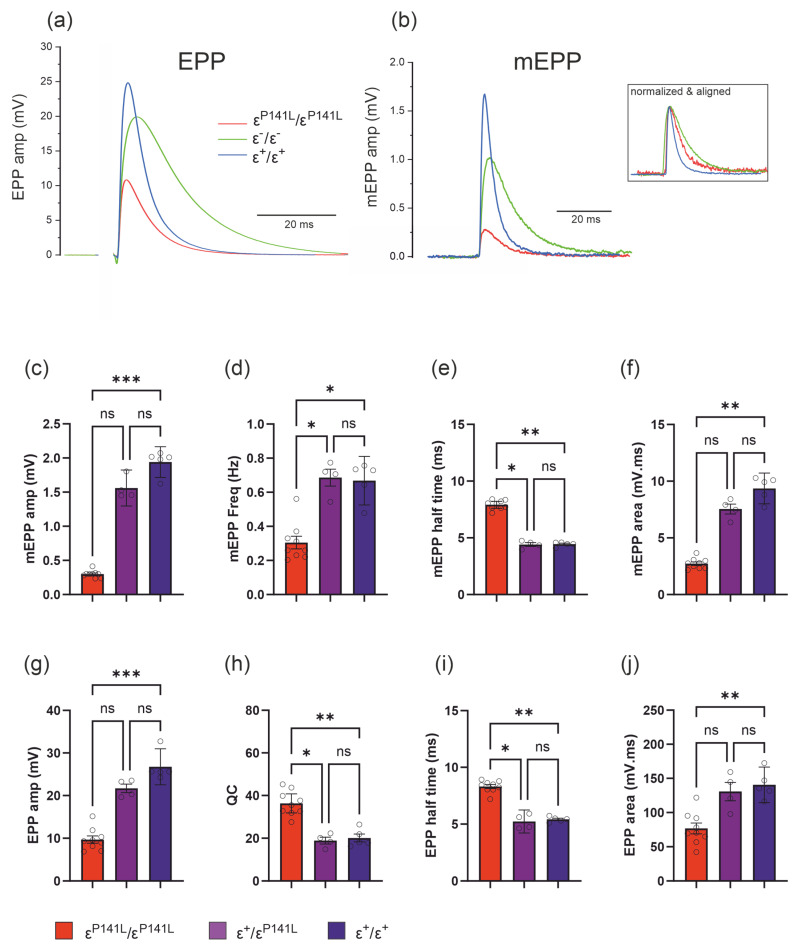
Example traces of diaphragm neurotransmission and quantification of 8 parameters of neurotransmission at post-natal days (PNDs) 15–17. Panel (**a**) shows example traces of endplate potentials (EPPs) and (**b**) miniature endplate potentials (mEPPs) from ε^P141L^/ε^P141L^, ε^+^/ε^+^ and ε^−^/ε^−^ diaphragms (averaged from a train of 20 stimuli at 1 Hz for EPPs and at least 20 spontaneous mEPPs). Inset shows mEPPs normalized and aligned to highlight differences in mEPP duration. Panels (**c**–**j**) show 8 parameters of neurotransmission recorded from the diaphragm from ε^P141L^/ε^P141L^ (*n* = 9), ε^+^/ε^P141L^ (*n* = 4) and ε^+^/ε^+^ (*n* = 5) mice at PNDs 15–17; the top row shows mEPPs and the bottom row shows EPPs. Significant differences are indicated; non-significant differences are not indicated. Kruskal–Wallis non-parametric ANOVA and Dunn’s multiple comparisons were used to determine significant differences. Each data point is the mean of all recordings from individual preparations (*n* = 6 to 16 fibers), overall means are indicated by bars, and error bars represent 95% confidence intervals. Statistical significance is indicated; ns *p* ≥ 0.05; * *p* < 0.05; ** *p* < 0.01; *** *p* < 0.001.

**Figure 3:**
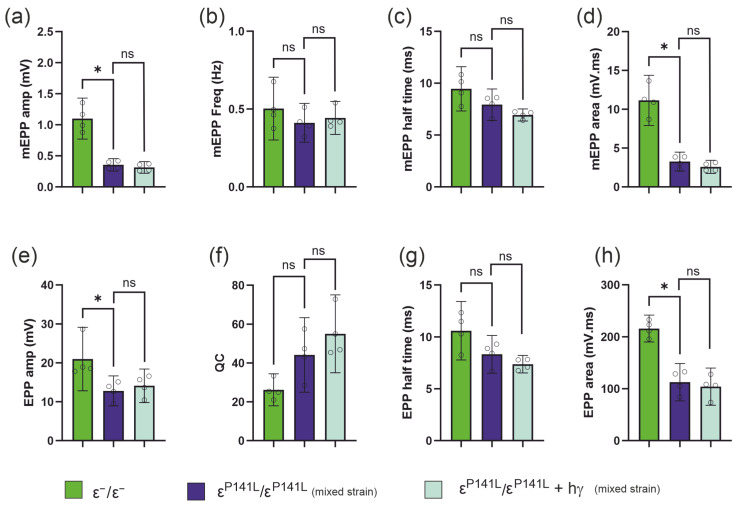
Comparison of 8 parameters of neurotransmission in diaphragm at PNDs 15–17 from ε KO mice and model mice additionally expressing hg. The panels show 8 parameters (**a**–**h**) of neurotransmission recorded from the diaphragm from ε^P141L^/ε^P141L^ (*n* = 4), ε^−^/ε^−^ (*n* = 4) and ε^P141L^/ε^P141L^ + hg (*n* = 4) mice at PNDs 15–17; the top row shows mEPPs and the bottom row shows EPPs. Non-parametric Mann–Whitney comparison tests of preparation means were used to determine significance. Each data point is the mean of all recordings from individual preparations (*n* = 11 to 15 fibers), overall means are indicated by bars, and error bars represent 95% confidence intervals. statistical significance is indicated; ns *p* ≥ 0.05; * *p* < 0.05.

At PND15 to PND17, neuromuscular transmission in phrenic nerve/diaphragm muscle preparations was assessed ex vivo (Figure 2). Spontaneous release of ACh from single pre-synaptic vesicles resulted in miniature endplate potential depolarizations (mEPPs), whilst phrenic nerve supra-threshold stimulation (at a 1 Hz train rate) generated a concerted release of multiple ACh-vesicles, which led to concerted endplate depolarization (EPP) sufficient to propagate muscle contraction (Figure 2a,b). Compared to age-matched WT littermates (ε^+^/ε^+^), fast-channel mice (ε^P141L^/ε^P141L^) had reduced mEPP amplitude, area and frequency, and reduced EPP amplitude and area, whilst the mEPP half time, EPP half time, and quantal content (QC, the number of vesicles released per stimulation) were all increased. No significant differences between male and female fast-channel mice were found (male *n* = 4, female *n* = 5) (Figure 2c–j). These changes to neuromuscular transmission parameters (particularly the increased duration of mEPPs compared to WT) are reminiscent of AChR-deficiency mouse models, suggesting the inclusion of γ subunits forming fetal channels, which exhibit alternative kinetic properties [20,21]. However, compared to age-matched epsilon knockout mice (ε^−^/ε^−^), fast-channel littermate mice had reduced mEPP amplitude and area, and reduced EPP amplitude and area (Figure 3a–h). These changes likely underlie the increased severity of fast-channel mice compared to age-matched AChR-deficiency mice.

To further investigate these observations, we additionally crossed ε^P141L^ mice with CHRNG-expressing [19] mice. Insertion of human *CHRNG* (ε^P141L^/ε^P141L^ + hγ) did not significantly change any parameter of neuromuscular transmission compared to age-matched ε^P141L^/ε^P141L^ littermate mice (Figure 3a–h). In summary, the addition of hγ did not seem to increase fetal AChR incorporation at the NMJ and did not significantly increase survival.

### 3.4. Pharmacology

**Figure 4:**
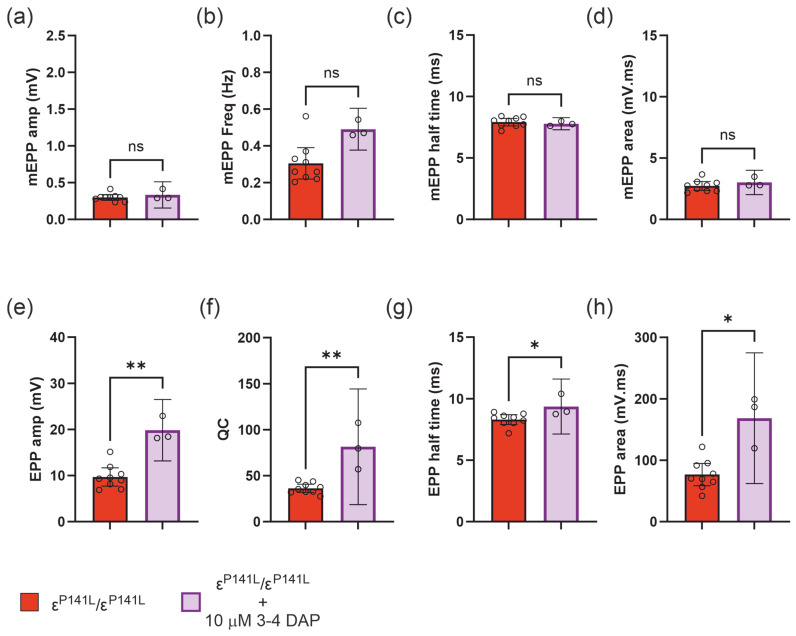
3-4 Diaminopyridine enhances neurotransmission in the diaphragm of ε^P141L^/ε^P141L^ mice at PNDs 15–17. The panels show 8 parameters (**a**–**h**) of neurotransmission recorded from the diaphragm from ε^P141L^/ε^P141L^ (*n* = 9) and ε^P141L^/ε^P141L^ with 10 µM 3,4-DAP (*n* = 3) added to the bathing solution from mice at PNDs 15–17; the top row is for mEPPs and the bottom row is from EPPs. Non-parametric Mann–Whitney comparison tests of preparation means were used to determine significance. Each data point is the mean of all recordings from individual preparations (*n* = 6 to 14 fibers), overall means are indicated by bars, and error bars represent 95% confidence intervals. Statistical significance is indicated; ns *p* ≥ 0.05; * *p* < 0.05; ** *p* < 0.01.

One of the current best frontline treatments for FCGMS patients is 3,4-DAP that acts on pre-synaptic voltage-gated potassium channels to enhance the release of ACh-containing vesicles [13]. 3,4-DAP (10 µM) was bath-applied to isolated phrenic-nerve/diaphragm preparations. Compared to untreated fast-channel mice (ε^P141L^/ε^P141L^), there was a significant increase in EPP amplitude, EPP half time, EPP area and QC. There was no change in mEPP amplitude, half time or area (Figure 4a–h). These changes in NMJ physiology would be expected as 3,4-DAP increases pre-synaptic ACh release to enhance neuromuscular transmission, but has no known effects on post-synaptic signal transduction.

**Figure 5:**
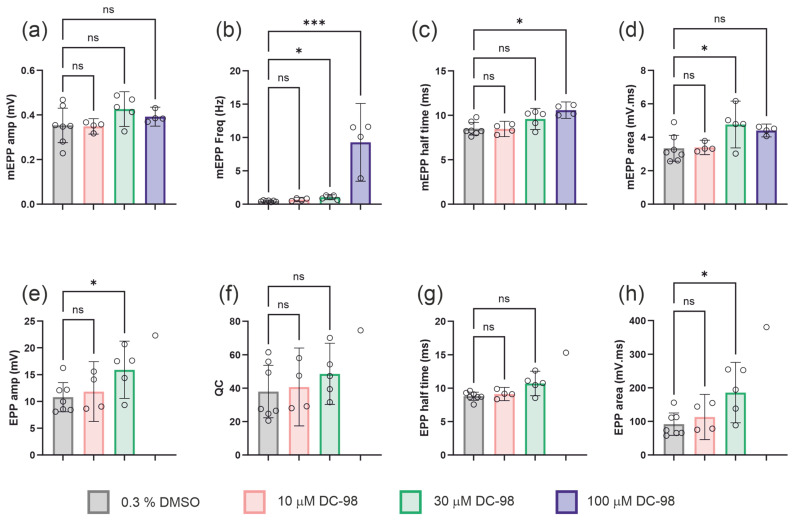
AChR PAM DC-98 enhances neurotransmission in the diaphragm of ε^P141L^/ε^P141L^ mice at PNDs 15–17. The panels show 8 parameters of neurotransmission (**a**–**h**) recorded from the diaphragm from ε^P141L^/ε^P141L^ mice at PNDs 15–17. For each group, diaphragms were pre-incubated with 0.3% DMSO (*n* = 7) and 10, 30 or 100 µM DC-98 (*n* = 3, 5 and 4); the top row shows mEPPs and the bottom row shows EPPs. At 100 µM DC-98, a µ-conotoxin GIIIB-insensitive muscle twitch prevented the recording of EPPs in 3 out of 4 preparations, and data from one preparation is indicated. Kruskal–Wallis non-parametric ANOVA and Dunn’s multiple comparisons to the 0.3% DMSO group were used to determine significant differences. Each data point is the mean of all recordings from individual preparations (*n* = 6 to 15 fibers); overall means of preparations are indicated by bars; and error bars represent 95% confidence intervals. Statistical significance is indicated; ns *p* ≥ 0.05; * *p* < 0.05; *** *p* < 0.001.

A potential novel treatment for FCGMS is an AChR-positive allosteric modulator DC-98-LC74 (DC-98 from now on) that we previously showed to increase the activity of εP141L mutant AChR [22]. Compared to 0.3% DMSO vehicle controls, mEPP amplitude was not significantly increased, although 30 µM did show a small enhancement (Figure 5). mEPP frequency was significantly increased when 30 or 100 µM was applied (Figure 5b). Similarly, mEPP half time was significantly increased at 100 µM (Figure 5c), and mEPP area was significantly increased at 30 µM DC-98 (Figure 5d). EPP amplitude was significantly enhanced at 30 µM DC-98 (Figure 5e). EPP area was significantly increased by 30 µM DC-98 (Figure 5h). Due to excessive tissue movement (likely due to enhanced pre-synaptic vesicle release), EPPs could only be recorded in 1 out of 4 preparations exposed to 100 µM DC-98.

## 4. Discussion

In this manuscript, we demonstrate that mice homozygous for εP141L have a severe phenotype, not surviving past weaning age, and are a reasonable facsimile of FCGMS caused by the homologous variant, where untreated patients do not survive childhood. This model has a significantly more severe phenotype than mice that express no ε-subunit whatsoever, suggesting that inclusion of mutant εP141L subunits is worse than having no ε-subunits at all. Confocal imaging showed robust α-bungarotoxin binding at the NMJ comparable to WT, with similar-sized AChR clusters that retained the classic pretzel-shaped structure. This is in stark contrast to the weak fragmented staining seen in AChR-deficiency mice, and corroborates previous reports of robust surface expression of εP141L-containing AChR in heterologous expression systems, with data from patient biopsies suggesting no defects in AChR expression or NMJ structure [14]. Further morphological characterization and quantification were not pursued, since no obvious staining defect was observed even in severely affected mice.

Previous detailed studies of εP141L AChR kinetic activity demonstrated a 280-fold decrease in activity, caused by the disruption of one of the ACh-binding sites at the α-ε interface [14,23], predicting a greatly abbreviated AChR endplate current. This should produce an unusually brief mEPP depolarization duration. However, the kinetics of the mEPPs recorded in the fast-channel mouse diaphragm have prolonged duration and appear more like γ-containing fetal channels, that have different kinetic properties to ε-containing adult channels, with prolonged activation [24,25]. We do not have direct evidence for inclusion of γ-containing fetal AChR; however, the time course of phenotypic worsening of model mice matches well with the expected loss of fetal AChR post-natally, and the relative lack of efficacy of DC-98 which does not modulate fetal AChR [22] and the prolonged endplate current duration are consistent with this hypothesis.

We cannot rule out that εP141L-AChR contributes to a small component of post-synaptic depolarization, but it appears to be overwhelmed or masked by the longer-duration fetal channel current. What is clear is that as the contribution of the fetal channel current diminishes through channel turnover, the remaining current is insufficient to support life. This occurs earlier than in ε^−^/ε^−^ mice, and the presence of the εP141L-AChR channel is more detrimental than having no adult channels whatsoever.

In mice, expression of the γ-subunit is stopped shortly after birth [26,27]. AChRs inserted at the NMJ have a limited half-life of ~8 days [28]; therefore, existing γ-containing fetal AChRs are eventually removed and degraded. This appears to coincide with disruption of neurotransmission and weight loss leading ultimately to death. For *Chrne* knockout mice, cross-breeding with a human γ-subunit knock-in strain (ε^−^/ε^−^ + hγ) produces mice with normal lifespans [19]. This cross-bred model more accurately resembles the human *CHRNE* deficiency syndrome as γ is expressed throughout our lives [29], unlike mice who stop expressing γ shortly after birth [26,27]. However, cross-breeding fast-channel mice with the same human γ-subunit knock-in strain (ε^P141L^/ε^P141L^ + hγ) only gave a 2-day enhancement in survival. This disparity in the effect of γ expression is consistent with the clinical phenotype of patients with these genotypes. Whereas *CHRNE*-deficient patients have relatively mild and stable myasthenia, εP141L-FCGMS patients are some of the most severe GMS patients, who do not survive childhood without treatment. As the level of γ expression at the NMJ was not determined in our mice, we cannot fully discount the potential benefit of over-expression of γ subunits in this model, but the enhanced survival of ε^−^/ε^−^ mice, which only lived for a median of 51 days in this study and others [30,31], would suggest that sufficient expression can occur. We hypothesize that this is due to the preferential incorporation of the εP141L subunit containing AChR, causing faster removal of the more functional γ-subunit containing fetal AChR from the NMJ post-synaptic membrane. This is possibly a result of the local high expression of ε-subunits by sub-synaptic nuclei [32], compared to the dwindling expression of γ-subunits, turned-off in post-natal life [26,27].

The mechanisms governing AChR pentamer assembly and the dynamics of subunit incorporation remain poorly understood. Whether localized over-expression of ε-subunits (enhanced by neural activity and neural Agrin deposition [33]) overwhelms the expression-restricted γ-subunit, or whether there is an assembly preference for inclusion of the ε-subunit that cannot be overcome even by over-expression of the γ-subunit, is not known. Additionally, it is unknown whether there are differences in the mechanisms of incorporation of adult and fetal receptors into the post-synaptic membrane, or whether there are differences in their stability. The ε^P141L^ mouse model may provide a valuable platform and experimental system for investigating these important questions.

Preliminary studies testing the treatment of diaphragms from fast-channel mice with 3,4-DAP produced the expected increase in pre-synaptic ACh-containing vesicle release, with increased mEPP frequency and QC. Enhancement reached levels similar to previous reports on AChR-deficiency model mice (ε^−^/ε^−^ + hγ), compatible with enhanced longevity [19]. However, as much of the neuromuscular transmission is carried out by residual fetal AChR, which will diminish as the mice continue to develop, we would also expect the response to 3,4-DAP to reduce until all endogenous *Chrng* is lost. Only at this point will we see the full extent of the fast-channel phenotype, and how resistant it is to current treatments.

Overall, in preliminary studies the effects of the positive allosteric modulator (PAM) DC-98 on EPP area at 30 µM were similar to those of 3,4-DAP at 10 µM (185.5 ± 32.4 mV.ms vs. 168.7 ± 24.7 mV.ms, *n* = 5 and 3, *p* = 0.36). The largest changes caused by DC-98 (on mEPP frequency and EPP amplitude) were on pre-synaptic functional parameters not related to action on post-synaptic AChR, with limited effect on mEPP area at lower doses, and not replicating the mEPP area observed in WT preparations (4.76 ± 0.5 vs. 9.36 ± 0.5 mV.ms, *n* = 5 and 5, *p* < 0.0001). Our previous work showed that DC-98 does not modulate γ-containing AChR; therefore, the small action of the compound, seen in this preliminary small-scale study, is possibly due to prolongation of a minimally contributing εP141L-AChR current fraction. This interpretation is complicated by the “off-target” effects of DC-98, which seem to enhance pre-synaptic vesicular release characteristics, which can contribute to enhanced EPP amplitude/duration and QC. The greater effects observed at 100 µM DC-98 suggest that the effect of the molecule is limited by low potency, and development of more potent PAMs may yield a more therapeutic response.

## 5. Limitations of the Study

Due to the early death of fast-channel model animals, strength tests and electromyography were not carried out, because of the technical challenges. This also meant we could only carry out a limited pharmacology study with limited numbers, which was only conducted on ex vivo phrenic nerve/diaphragm preparations. Rescue of the severe phenotype of ε^P141L^/ε^P141L^ mice by inclusion of γ-containing fetal AChR was not rigorously assessed, as γ expression was not quantified, so insufficient over-expression may have limited the effectiveness. Future studies are required to examine the level of γ-subunit expression and incorporation into the NMJ, and how this changes over time.

## 6. Conclusions

Characterization of C57BL/6J-Chrne^em1H^/H mice with the *Chrne* p.P141L variant knocked-in showed that it recapitulates the severe FCGMS phenotype of patients with the homologous variant and demonstrates why these patients are more severely affected than those with no *CHRNE* expression. We also show in ex vivo electrophysiology studies that 3,4-DAP and the muscle AChR PAM DC-98 improved neuromuscular transmission. Thus, this model is a valuable tool both for understanding the pathogenic mechanism of FCGMS, and as a platform for developing new treatments for this severe form of genetic myasthenia.

## Data Availability

The original contributions presented in this study are included in the article/Supplementary Materials. Further inquiries can be directed to the corresponding author. C57BL/6J-Chrne^em1H^/H mice are available from Mary Lyon Centre, MRC Harwell.

## Supplementary Materials

The following supporting information can be downloaded at https://www.mdpi.com/article/10.3390/biom16070931/s1, Figure S1: Examples of NMJ AChR staining and morphology.

## Abbreviations

3,4-DAP	3,4-Diaminopyridine
AChR	Acetylcholine Receptor
Chrne	Mouse Muscle Acetylcholine Receptor Epsilon Subunit Gene
CHRNG	Human Muscle Acetylcholine Receptor Gamma Subunit Gene
GMS	Genetic Myasthenic Syndrome
EPP	Endplate Potential
FCGMS	Fast-Channel Genetic Myasthenic Syndrome
mEPP	miniature Endplate Potential
NMJ	Neuromuscular Junction
PAM	Positive Allosteric Modulator
pLGIC	pentameric Ligand-Gated Ion Channel
PND	Post-Natal Day
QC	Quantal Content
QMG	Quantitative Myasthenia Grading
