# Supplementary material for: Mouse Model of Fast-Channel Genetic Myasthenic Syndrome Carrying Chrne p.P141L Mutation
Source: Biomolecules. 2026 Jun 23;16(7):931. doi: 10.3390/biom16070931 (PMC13406490; doi:10.3390/biom16070931)
Supplement: Supplementary file 1 [file biomolecules-16-00931-s001.zip › biomolecules-4305704-supplementary.pdf]

Supplementary material

Generation of C57BL/6J-Chrne[em1H]/H mice

Name of Mouse model or mutation:

Chrne-P141L-EM1-B6J

Description:

Point mutation made by CRISPR/Cas9 gene editing.

Type of mutation:

SNP: P141L

Delivery method:

Electroporation into 1-cell stage embryo.

Genetic Background:

C57BL/6J

Nuclease:

90:10 Deactivated Cas9: Cas9 protein

sgRNAs:

Protospacer sequence	PAM sequence
AGGTGCTGCGGTAGATGGCT	GGG

ssODN donor sequence (5′-3′):

ATGTGAAAAGCTTGGTTTCCACAGTATTGATGGGCAGTTTGGAGTGGCCTACGACAGCAAT
GTTCTAGTCTATGAGGGAGGCTATGTGAGCTGGTTGCCaCtAGCCATCTACCGCAGCACCTG
CGCAGTGGAGGTCACCTATTTCCCCTTTGACTGGCAGAACTGCTCTCTCATTTTTCGGTAGG
AAGAATCACTTCAAT

Electroporation mixes:

90:10 Deactivated Cas9: Cas9 protein, sgRNAs and ssODNs were diluted and mixed in Electroporation buffer (EB; Gibco Opti-MEM I Reduced Serum Media – (Thermo Fisher Scientific)) to the working concentrations of 650 ng/µl, 130 ng/µl and 400 ng/µl, respectively. Embryos were electroporated using the following conditions: 30 V, 3 ms pulse length, 100 ms pulse interval, 12 pulses. Electroporated embryos were re-implanted in CD1 pseudo-pregnant females. Host females were allowed to litter and rear F_0 progeny.

Sequence details

Chrne WT

ATAGGCAGAAGCCAGGGAAAGAATGAAGAGCTTAGCCTGTATCACCATCT
CTTCGACAATTATGATCCAGAATGCCGGCCAGTTAGGAGACCTGAGGACA
CTGTCACCATCACCCTCAAGGTCACCCTAACCAACCTCATCTCACTGGTAA
GACCTTCCCCCAATTCTAATCCTATACCCTCACTTCTACCCCCTAACTCCTG
GCTTCAGCAGGCTTCTTTCTGTTGTAGAACGAGAAAGAAGAAACTCTGACC
ACCAGTGTCTGGATTGGCATTGTGAGTCAGACCTGGGGAGCAGTGGGAGG
GTCCCCAGCCAAGCCCCTTCTCTTCTGCCAGGTGGGAGCCGGAGAGCACAC
ACTCTCTCTCGCCCCCACCTCTTAGGACTGGCACGACTATCGGCTCAACTAC
AGCAAGGACGATTTTGCAGGTGTAGGAATCCTCCGGGTCCCTTCAGAACAT
GTATGGCTGCCAGAGATTGTTCTAGAAAACAAGTGAGGATCACCCAAGAG
GGCAGGCTGCAAAGGGAACTGGCTTTGGGGGATTTGGTTGGTGAAGGAGG
TTCAGACATGTGAAAAGCTTGGTTTCCACAGTATTGATGGGCAGTTTGGAG
TGGCCTACGACAGCAATGTTCTAGTCTATGAGGGAGGCTATGTGAGCTGGT
TGCCCCCAGCCATCTACCGCAGCACCTGCGCAGTGGAGGTCACCTATTTCC
CCTTTGACTGGCAGAACTGCTCTCTCATTTTTCGGTAGGAAGAATCACTTCA
ATCATGATATTGAGAAGGGCCTAGAGCAGGGGACAGGGCCTGCTAAGGAG
GGATAGGTGGGGTCTTCTTTGGAAGGTCTTTGGGAAGGAGTTGGAGTGAGG
CACTGGAGTGAAGGGGTGGGAAGCTGGTGTAGGTTTGCAGTAGGCGTGGG
CACAGGTTTCTTGCTGCCATCTTGTAGTTTGAGCGCATAGTCGCAGCTATTC
CCCAGGAGGAGTCTGAGGCATAAAAATTACTTGAGTCCAGGAATCCCTTG
GATAGCACGGTAAGCCCATCAGCAACCCAAAACAGAACAGAAAAGGAAG
CAGGATTGGGAACGCGCTGGGGAATGTGGGCAAAAGAAGAGAGCCATATT
ACCCTGCCAGATTGAAGCTTCCAGGGTGGAACTAAGATGGCTGCCAGCTTC
TGGGGTAAAGACTGGCCCTGTTTCACAGCTCCCAGACCTACAATGCTGAGG
AGGTGGAGTTCATCTTTGCCGTGGATGACGACGGCAATACCATCAACAAG
ATTGACATTGACACGGCAGCTTTTACCGGTGAAGGGATATACCCCCTTTCC
AAAAATTCTTCTCCCAGATAAGGTTCAAGGGGGGACGTTCAGGGTAGGGCT
TGGCCTGAACCACACAACCGGAAG

Chrne-P141L-EM1-B6J
ATAGGCAGAAGCCAGGGAAAGAATGAAGAGCTTAGCCTGTATCACCATCT
CTTCGACAATTATGATCCAGAATGCCGGCCAGTTAGGAGACCTGAGGACA
CTGTCACCATCACCCTCAAGGTCACCCTAACCAACCTCATCTCACTGGTAA
GACCTTCCCCCAATTCTAATCCTATACCCTCACTTCTACCCCCTAACTCCTG
GCTTCAGCAGGCTTCTTTCTGTTGTAGAACGAGAAAGAAGAAACTCTGACC
ACCAGTGTCTGGATTGGCATTGTGAGTCAGACCTGGGGAGCAGTGGGAGG
GTCCCCAGCCAAGCCCCTTCTCTTCTGCCAGGTGGGAGCCGGAGAGCACAC
ACTCTCTCTCGCCCCCACCTCTTAGGACTGGCACGACTATCGGCTCAACTAC
AGCAAGGACGATTTTGCAGGTGTAGGAATCCTCCGGGTCCCTTCAGAACAT
GTATGGCTGCCAGAGATTGTTCTAGAAAACAAGTGAGGATCACCCAAGAG
GGCAGGCTGCAAAGGGAACTGGCTTTGGGGGATTTGGTTGGTGAAGGAGG
TTCAGACATGTGAAAAGCTTGGTTTCCACAGTATTGATGGGCAGTTTGGAG
TGGCCTACGACAGCAATGTTCTAGTCTATGAGGGAGGCTATGTGAGCTGGT
TGCC**a**C**t**AGCCATCTACCGCAGCACCTGCGCAGTGGAGGTCACCTATTTCCC

CTTTGACTGGCAGAACTGCTCTCTCATTTTTCGGTAGGAAGAATCACTTCAA
TCATGATATTGAGAAGGGCCTAGAGCAGGGGACAGGGCCTGCTAAGGAGG
GATAGGTGGGGTCTTCTTTGGAAGGTCTTTGGGAAGGAGTTGGAGTGAGGC
ACTGGAGTGAAGGGGTGGGAAGCTGGTGTAGGTTTGCAGTAGGCGTGGGC
ACAGGTTTCTTGCTGCCATCTTGTAGTTTGAGCGCATAGTCGCAGCTATTCC
CCAGGAGGAGTCTGAGGCATAAAAATTACTTGAGTCCAGGAATCCCTTGG
ATAGCACGGTAAGCCCATCAGCAACCCAAAACAGAACAGAAAAGGAAGC
AGGATTGGGAACGCGCTGGGGAATGTGGGCAAAAGAAGAGAGCCATATT
ACCCTGCCAGATTGAAGCTTCCAGGGTGGAACTAAGATGGCTGCCAGCTTC
TGGGGTAAAGACTGGCCCTGTTTCACAGCTCCCAGACCTACAATGCTGAGG
AGGTGGAGTTCATCTTTGCCGTGGATGACGACGGCAATACCATCAACAAG
ATTGACATTGACACGGCAGCTTTTACCGGTGAAGGGATATACCCCCTTTCC
AAAAATTCTTCTCCCAGATAAGGTTCAAGGGGGGACGTTCAGGGTAGGGCT
TGGCCTGAACCACACAACCGGAAG

Red highlight and bold = nominated change. **Yellow highlight and bold = silent mutation** to prevent the reprocessing of the engineered allele.

Heterozygous F1 animal sequence trace:

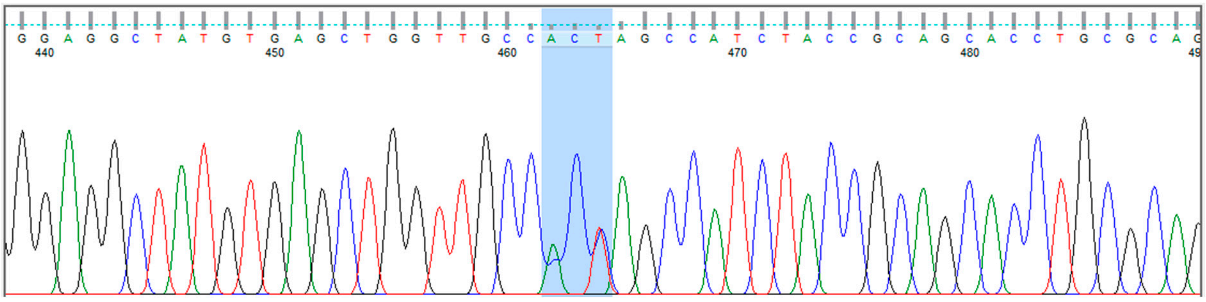

Nucleotide Alignment:

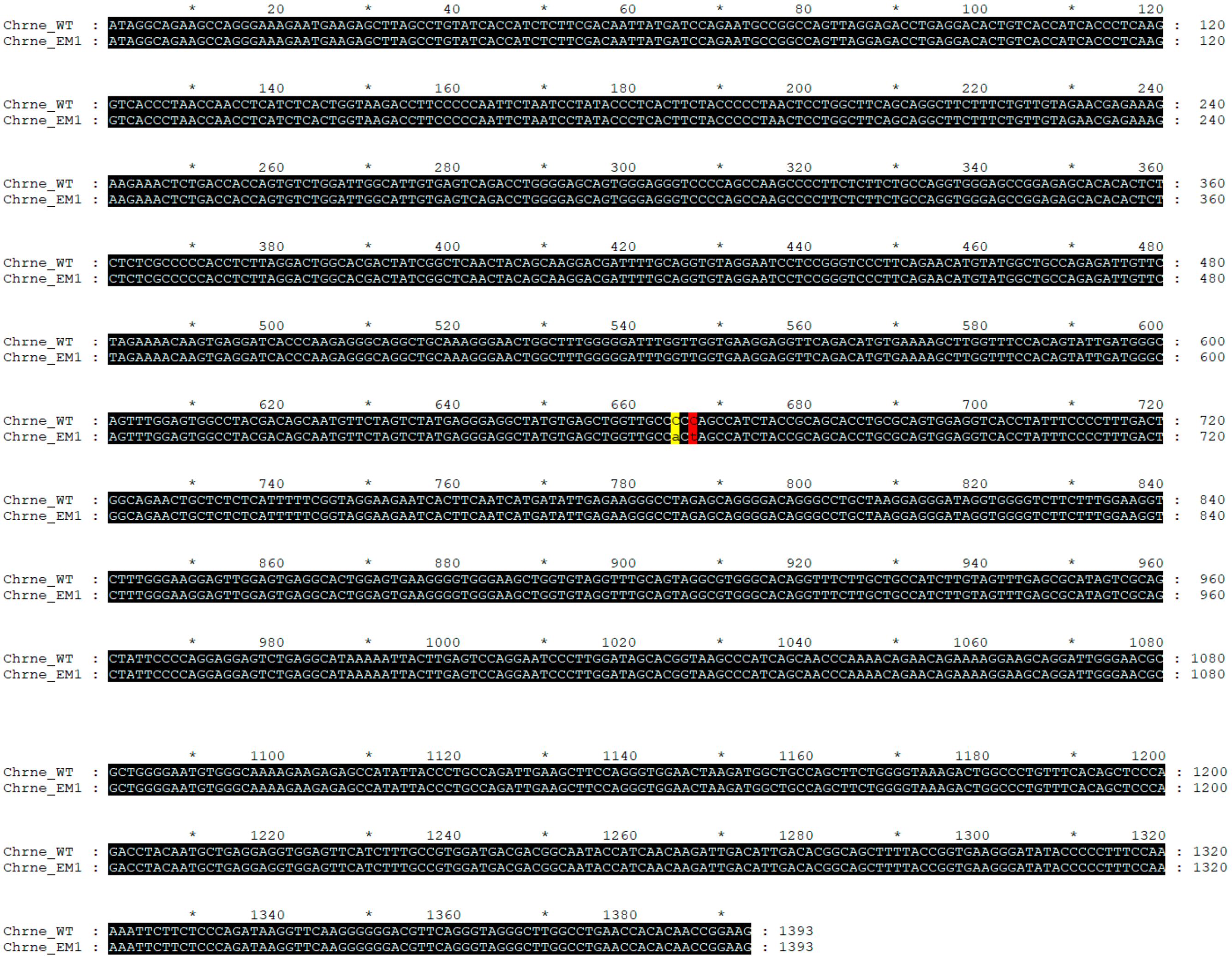

Predicted Protein Alignment:

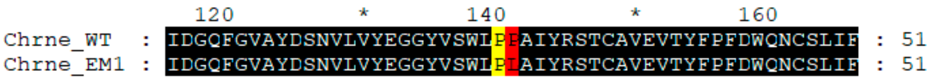

QC strategy employed at Harwell to check the edited allele:

Genomic DNA was extracted from ear clip biopsies and amplified in a PCR reaction using the following conditions/primer sequences:

Geno_Chrne_P141L_F1 (5' to 3')	ATAGGCAGAAGCCAGGGAAAG
Geno_Chrne_P141L_R1 (5' to 3')	CTTCCGGTTGTGTGGTTCAGG
Taq Polymerase used	ThermoFisher SuperFi II PCR Kit
Annealing Temperature (°C)	60
Elongation time (min)	1
WT product size (bp)	1393
Mutant product size (bp)	1393
Notes	Please sequence with the following primers (5′ to 3′):<br>Geno_Chrne_P141L_F2:<br>ATACCCTCACTTCTACCCCCT<br>Geno_Chrne_P141L_R2: GCAGCCATCTTAGTTCCACC

All amplicons were sent for Sanger sequencing to check for integration of the donor oligo sequence at the target site. F1 sequences should be heterozygous unless on sex chromosome.

Off-target site with ≤2 mismatches for guide(s) used were checked with the following primers:

Off-target site	Sequence	Type	Primers used (5′-3′)
11:99082178-99082200	**TT**GT**C**CTG**A**GGTAGATGGCT GGG	Intronic	Chrne_P141L_OT4F1: TCGGTGACTGGAGATTGTGTG Chrne_P141L_OT4R1: TGTGTGTCCCTGTGGCTAAG
11:102659253-102659275	AGGTG**GCTT**GGTAGATGGCT TGG	Intergenic	Chrne_P141L_OT5F1: AGGCATGCTTGGCTCTTATG Chrne_P141L_OT5R1: TTGCTAAACATGGTGCTGGC<br><br>Please sequence with the following primer: Chrne_P141L_OT5R2: GCTTCGCAATAAGGAACGCA
11:63982834-63982856	AG**C**TGCTG**T**GGTAGA**G**GGCT GGG	Intronic	Chrne_P141L_OT6F1: ACATGAGCTGTGCCTAACCC Chrne_P141L_OT6R1: CTTGGCAGGGAGGTGAGAAG

All amplicons were sent for Sanger sequencing.
No off-target activity was detected in the animals selected to establish the colony.

Additional integrations of the donor sequence

Copy counting of the donor sequence was carried out by ddPCR at the F1 stage to confirm donor oligos were inserted once on target into the genome. The following Taqman assay was used to copy count the donor sequence compared against a VIC-labelled reference assay for Dot1l:

Assay name	Chrne_P141L_MUT1
Forward Primer (5′-3′)	GTGAGCTGGTTGCCACT
Reverse Primer (5′-3′)	AGCAGTTCTGCCAGTCAAA
Probe (5′-3′)	TGCGCAGTGGAGGTCACCTATTTC
Label	FAM

This ddPCR assay is specific to the donor used to create the engineered mutation and only mutant alleles are expected to be recognised by this assay. Therefore, WT controls are expected to call at 0 copies and a single integration for a correct mutation is expected to call at 1 copy for F1 (HET) animals.

Reference Assay Name	Dot1l
Forward primer (5′-3′)	GCCCCAGCACGACCATT

Reverse primer (5′-3′)	TAGTTGGCATCCTTATGCTTCATC
Probe (5′-3′)	CCCAACAGGCCTGGATTCTCAATGC
Label	VIC

VIC-labelled reference assay for Dot1l gene.

No additional donor integrations were detected in the animals taken forward to establish the colony.

Official nomenclature (to be used in publications): C57BL/6J-Chrne^{em1H}/H

Repository ID: EM:16095

Origin of strain: This strain carries a point mutation (P141L) introduced into the gene Chrne by CRISPR/Cas9 gene editing.

Coat Colour: Non-agouti (Black)

Breeding recommendation: Maintain on a C57BL/6J background

Mice used in this study were obtained from the Mary Lyon Centre at MRC Harwell (MLC) and the following award is acknowledged: MC_UP_2201/2.

The mouse model was generated in collaboration with Genome Editing Mice for Medicine Programme at Mary Lyon Centre MRC Harwell (https://www.har.mrc.ac.uk/projects/gemm/).

The C57BL/6J-Chrne^{em1H}/H mice were obtained from the Mary Lyon Centre at MRC Harwell which is the UK node of the European Mouse Mutant Archive (EMMA) (https://www.infrafrontier.eu/emma/strain-details/?q=16095)

Fig S1

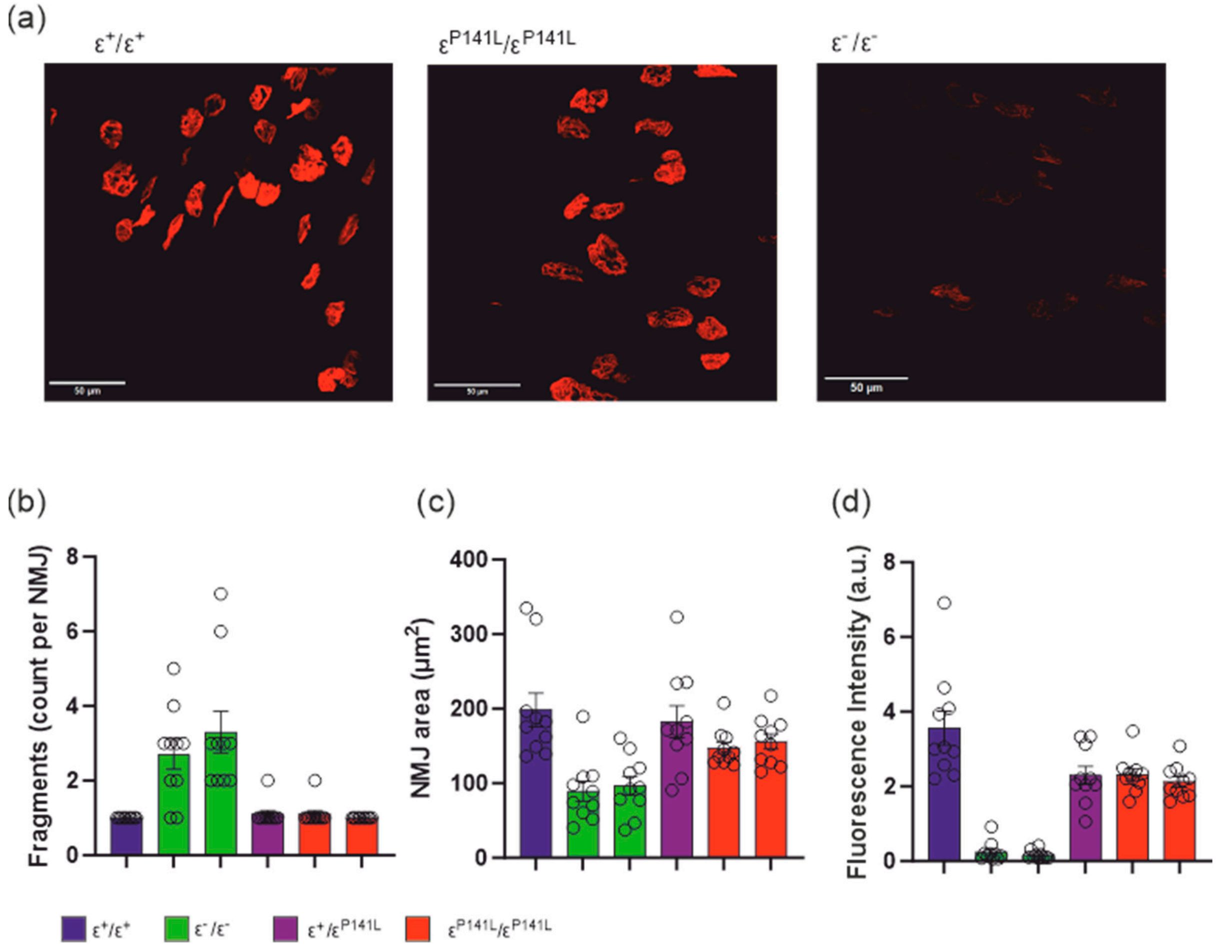

Figure S1. Examples of NMJ AChR staining and morphology (a) Example images of AChR stained with α-bungarotoxin (594 nm fluorescence) of multiple NMJ in PND 15-17 diaphragm muscle from $\varepsilon^{+}/\ \varepsilon^{+}$, $\varepsilon^{P141L}/\ \varepsilon^{P141L}$ and $\varepsilon^{-}/\ \varepsilon^{-}$ mice. Quantification of NMJ fragmentation (b) area (c) and fluorescence intensity (d) of NMJ visualized by 594 nm α-bungarotoxin staining of PND 15-17 diaphragm muscle. Each bar represents mean ± SEM of individual preparations, each data point is an analyzed identified NMJ. These data were collected from limited samples and are only representative, statistical analyses were not carried out.
